# Evaluating the Impact of Sample Irregularities on the Dynamic Stiffness of Polyurethane: Insights from Experimental and FEM Analysis

**DOI:** 10.3390/ma17235910

**Published:** 2024-12-03

**Authors:** Krzysztof Nering, Arkadiusz Kwiecień, Konrad Nering

**Affiliations:** 1 Faculty of Civil Engineering, Cracow University of Technology, 31-155 Cracow, Poland; 2 FlexAndRobust Systems Ltd., 31-155 Cracow, Poland; ak@flexandrobust.com; 3 Faculty of Mechanical Engineering, Cracow University of Technology, 31-155 Cracow, Poland; konrad.nering@pk.edu.pl

**Keywords:** dynamic stiffness, vibroacoustic testing, polyurethane, damping, sample irregularities, FEM modeling

## Abstract

This study investigates the dynamic stiffness and damping characteristics of three polyurethane materials—PM, PS, and PST—using a comprehensive vibroacoustic testing approach. The aim is to examine material parameters such as dynamic stiffness, Young’s modulus, critical damping factor, and the influence of sample irregularities on the accuracy of measurements. The study employs both experimental testing, in which cuboidal and cylindrical polyurethane samples were subjected to sinusoidal excitation, and finite element modeling (FEM) to simulate the test conditions in sample without irregularities. Results indicate that sample contact surface irregularities (even as low as ~0.04 mm) significantly impact the measured dynamic stiffness, with the effect intensifying for materials with higher Young’s modulus values (above 5 MPa). Furthermore, cylindrical samples demonstrated more stable and repeatable measurements compared to cuboidal samples, where surface irregularities were tested in a more controlled environment. The findings underscore the need to consider sample geometry and irregularities in dynamic stiffness assessments to ensure better material evaluations. This work contributes valuable insights for the accurate modeling and testing of materials used in vibration isolation and sound insulation contexts.

## 1. Introduction

The ongoing development of global economies is leading more people to move into urban areas. As a result, population density in cities is increasing. It is estimated that this trend will continue in developing countries [1,2,3,4]. In response, there has been growth in transportation infrastructure and the construction of multifamily housing [5,6,7,8,9,10]. However, one of the costs of this urbanization is a decrease in living comfort for residents in these areas. For the purposes of this study, it is important to emphasize the increased exposure to noise and transport-induced vibrations [11,12,13,14], as well as noise and vibrations from other residents in multifamily buildings and the same disruptive stimuli from building equipment [15,16,17,18]. There are specific guidelines aimed at mitigating this problem and improving the quality of life for residents [19,20,21]. However, to achieve this, it is essential to focus on the mechanics behind the emergence of these adverse effects [22,23,24].

The impact of noise on humans is well-researched. In addition to the obvious consequences, such as hearing damage from high noise exposure [25,26,27,28], there are other issues as well. Increased exposure to noise can lead to sleep disturbances, concentration problems, and general irritability [29,30,31,32]. It is particularly important to ensure comfortable conditions (quiet) during nighttime to support proper rest and the body’s recovery process.

Vibrations from transportation sources and their effects on the human body are also well-documented. Similar to noise, elevated levels of vibration affecting a person can lead to concentration and sleep disturbances [33,34,35,36]. It is worth noting that noise and vibrations, as disruptive stimuli, often occur together [37,38,39,40,41,42]. Therefore, it is beneficial to seek solutions that can simultaneously reduce both of these stressors.

Polyurethanes are materials that can be effectively used to control the emission or transmission of noise and vibrations [43,44,45]. They are commonly applied in vibration isolation for tram tracks [46,47,48,49], industrial machinery [50,51,52,53], and building elements in multifamily residential buildings [54,55,56,57,58]. Their excellent damping properties, wide range of stiffness options, and high durability allow them to be tailored for various applications. Polyurethane materials are characterized by versatile chemical networks, combining hard and soft segments that contribute to their dynamic mechanical properties, such as damping and stiffness. These properties are highly dependent on the polymer’s microstructure, phase separation, and crosslinking density, which play a crucial role in vibration and noise control applications. Recent studies have explored advanced polyurethane formulations and dynamic materials, emphasizing their tunable stiffness and viscoelastic behavior under varying conditions, also making them ideal candidates for sound damping systems [59,60,61,62].

The key to selecting the right material for a given solution lies in understanding its parameters. For instance, to accurately assess airborne and impact sound insulation between rooms, the critical parameter is dynamic stiffness [63,64,65,66,67,68], which can be determined according to ISO standard [69]. Airborne and impact sound insulation can also be estimated using computational methods like the Finite Element Method (FEM) [70,71,72,73]. In such cases, knowledge, at least, of Young’s modulus, Poisson’s ratio, and damping is essential. These parameters are also required to develop models that fine-tune vibration isolation systems to reduce the impact of vibrations from various sources on building residents [74,75,76,77].

Determining parameters through experimental methods always involves some degree of error [78,79]. These errors may arise from imperfections or limitations of the method or measuring equipment, as well as from improperly prepared material samples. In the case of noise and vibration control, protection against these stimuli, as mentioned before, primarily relies on tuning vibro-acoustic insulation to ensure the highest possible level of protection [80,81]. Underestimating the Young’s modulus can lead to using insulation that is too rigid, which may transmit noise or vibrations at excessively high frequencies. Overestimating the Young’s modulus, on the other hand, can result in overly compliant vibration isolation, causing excessive displacements within the isolated system, which may lead to damage (e.g., excessive rail deflection in a tram track) [82,83].

While previous studies have investigated the dynamic stiffness of polyurethane materials and their applications in sound and vibration isolation, few have systematically explored the influence of sample irregularities and geometry on dynamic stiffness measurements. For instance, prior work has primarily focused on idealized conditions, overlooking the practical challenges posed by surface irregularities and sample geometry variations [17,69,84,85,86]. In contrast, this study provides a comprehensive analysis of these factors, combining experimental and numerical methods to quantify their impact. The proposed shift from cuboidal to cylindrical sample geometries represents a novel approach to mitigating measurement variability caused by irregularities, offering practical insights for improving material characterization.

The novelty of the article lies in highlighting the scale of the issue related to underestimating dynamic stiffness (Young’s modulus) for rigid polyurethanes used in vibration isolation (Young’s modulus > 5 MPa), attributed to surface irregularities (up to 1 mm) in standard cuboidal samples. An approach was undertaken to mitigate this issue by altering the shape and size of the sample to a cylindrical form. Additionally, the consequences of introducing an increasing number of minor irregularities (up to 0.1 mm) in real-world sample conditions were simulated. A comparison was also made between the dynamic stiffness obtained from FEM modeling on cylindrical samples and ISO-based testing [69] on cuboidal samples, revealing significant discrepancies. These discrepancies clearly demonstrate the apparent softening of the material when a large design contact surface is affected by surface irregularities.

## 2. Methodology

**Figure 1:**
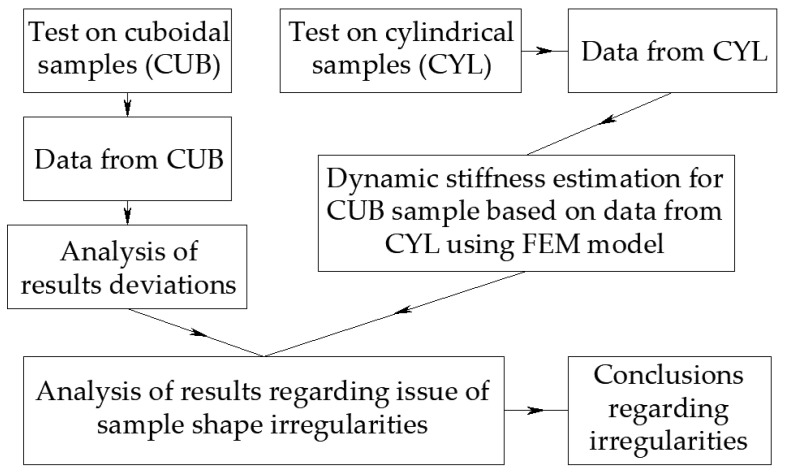
The research plan for this article.

In this section, the methodology of conducting the research is presented. The study is divided into two segments. The first segment concerns the determination of dynamic stiffness based on ISO 9052-1 [69], with material damping [69] based on [17,84,85] material samples compliant with this standard. The second segment involves determining dynamic stiffness with damping on cylindrical samples. The analysis of the results obtained from both segments aims to draw conclusions regarding the influence of irregularities in the tested samples on the dynamic stiffness result and related quantities. The article concept diagram is presented in Figure 1.

### 2.1. Test Equipment and Procedure

Equipment used for this test was a dynamic stiffness bench that is compatible with ISO standard [63]. The devices used to build the test bench have been listed in Table 1.

The testing apparatus was dynamically stimulated using a sinusoidal force generated by the exciter. This applied sinusoidal force had its amplitude consistently maintained at 0.4 N, with a tolerance of ±0.005 N. The frequency ranged from 20 Hz to 350 Hz, increasing by 0.1 Hz every second during the measurement period. An IEPE accelerometer mounted on the load plate recorded the system’s response.

**Figure 2:**
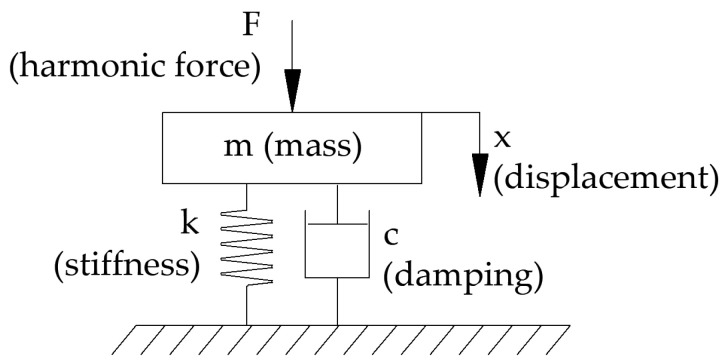
Single degree of freedom model as approximation of test bench.

The physical model of the test bench is shown in Figure 2.

This bench helps to provide information about the frequency response spectrum of the measured system. Based on the response spectrum, the resonant frequency of the tested system and the critical damping factor are determined. Based on the resonant frequency, the dynamic stiffness is calculated using the Equation (1)

(1) $$DS=4\pi^{2}m\prime f_{r}^{2}$$

where *DS*—dynamic stiffness [MN/m^3^], *m*’—mass per unit area in test [kg/m^2^], and *f_r_*—resonant frequency [Hz].

**Figure 3:**
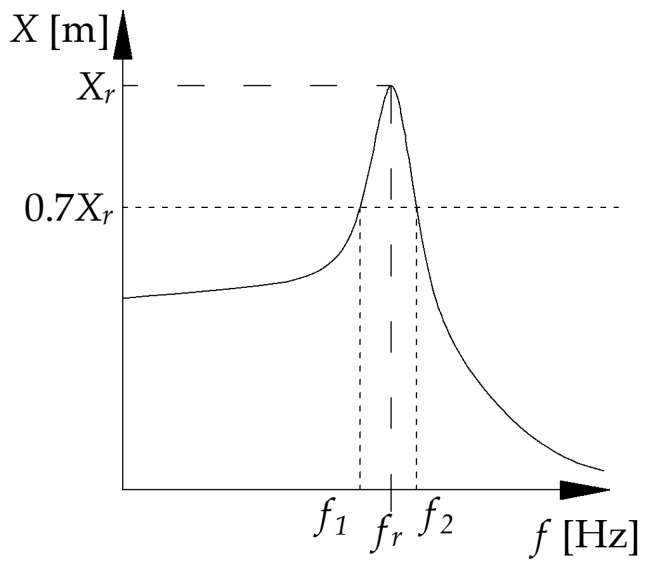
Schematic illustration of the half-power bandwidth method using the displacement spectrum. Here, X_r_ denotes the displacement amplitude at the resonant frequency f_r_, and f_1_ and f_2_ are the frequencies corresponding to 0.7 of the resonance amplitude.

The damping value was estimated using the half-power bandwidth method implemented by authors in MATLAB software (R2024a Update 1 (24.1.0.2568132)). However, to obtain the appropriate response spectrum, it must first be recalculated. The data obtained directly from the measurement are the acceleration values of vibrations from the accelerometer. In order to properly estimate the damping, the acceleration response spectrum should be transformed into the pseudo-displacement response spectrum (see Figure 3). This is achieved using Equation (2)

(2) $$\left|X(f)\right|=\frac{\left|A\left(f\right)\right|}{4\pi^{2}f^{2}}$$

where |*X*(*f*)|—pseudo-displacement spectrum [m], |*A*(*f*)|—acceleration spectrum [m/s^2^], and *f*—frequency [Hz].

The concept of extracting measured data from single degree of freedom mechanism is shown in Figure 3.

The critical damping factor (D) is obtained based on Equation (4) using the relationship described in Equation (3):

(3) $$\frac{f_{2}-f_{1}}{f_{r}}=\frac{\delta }{\pi \sqrt{1-{\left(\frac{\delta }{2\pi }\right)}^{2}}}$$

(4) $$\delta =\frac{2\pi D}{\sqrt{1-D^{2}}}$$

**Figure 4:**
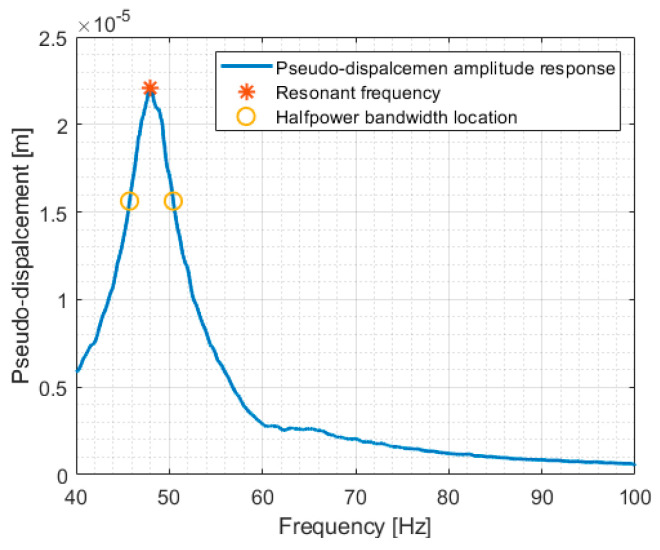
Exemplary result for PST cylindrical sample set with 5 pcs. f_r_ = 48.1 Hz.

Exemplary results of pseudo-displacement spectrum analysis are shown in Figure 4.

### 2.2. Material and Test Samples

The materials used in the research are polyurethanes PM, PS, and PST. These designations represent the trade names of products from FlexAndRobust Systems Ltd. (Cracow, Poland). These polyurethanes are solvent-free, elastic, two-component materials [87]. The samples for testing based on ISO [69] are rectangular specimens with dimensions of 200 mm × 200 mm and a height of 20 mm. Samples for simulating irregularities are cylindrical specimens with a diameter of 29 mm and a nominal height of 92 mm. Material declared values are shown in Table 2.

A summary of the samples along with their dimensions is presented in Table 3. Table 3 shows the average values of given parameters. In brackets, 95% credibility interval values are given. Samples were tested in following external conditions (average value with 95% credibility interval): Cuboid samples T = 25 °C (CI95%, 24.2; 25.7) °C, P_atm_ = 999 hPa (CI95%, 989; 1010) hPa, RH = 65% (CI95%, 60; 67)%, Cylindrical samples T = 23.5 °C (CI95%, 23.1; 23.9) °C, P_atm_ = 1001 hPa (CI95%, 994; 1005) hPa, RH = 44% (CI95%, 41; 52)%.

The measurements of the height and diameter of the samples were conducted using a micrometer screw gauge (accuracy of 0.01 mm). The results presented in Table 3 are derived from statistical analysis (mean and credibility interval). Higher precision was intentionally displayed to emphasize the small differences in the dimensions of the samples.

**Figure 5:**
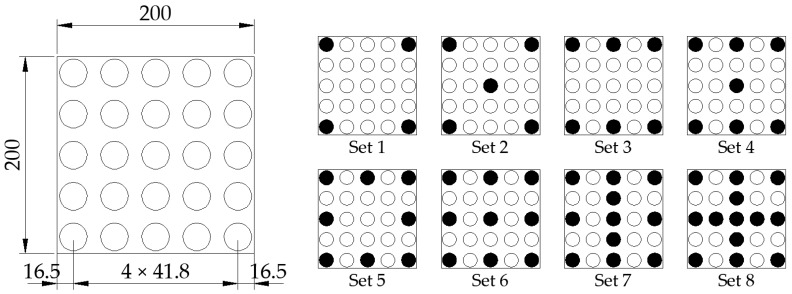
Diagram of cylindrical sample set for test.

The flat samples were tested individually (one sample per test). The cylindrical samples were tested in various configurations, with each batch containing between 4 and 13 samples in 3 batches. The layout diagram of the sample arrangement is shown in Figure 5.

**Figure 6:**
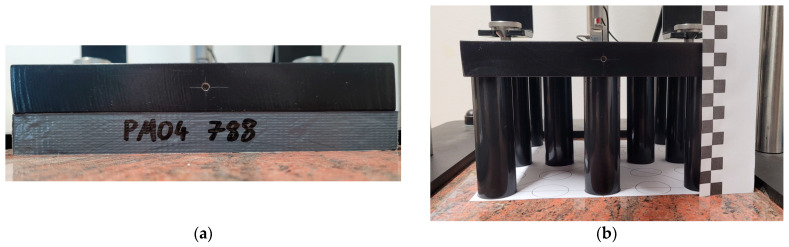
Test setup in real life for (**a**) cuboidal sample, (**b**) cylindrical samples (13 pcs. set).

An example test setup for a flat cuboidal sample and cylindrical samples is shown in Figure 6.

Each of the cuboidal samples had irregularities on its surface. A summary of these irregularities is presented in Table 4.

**Figure 7:**
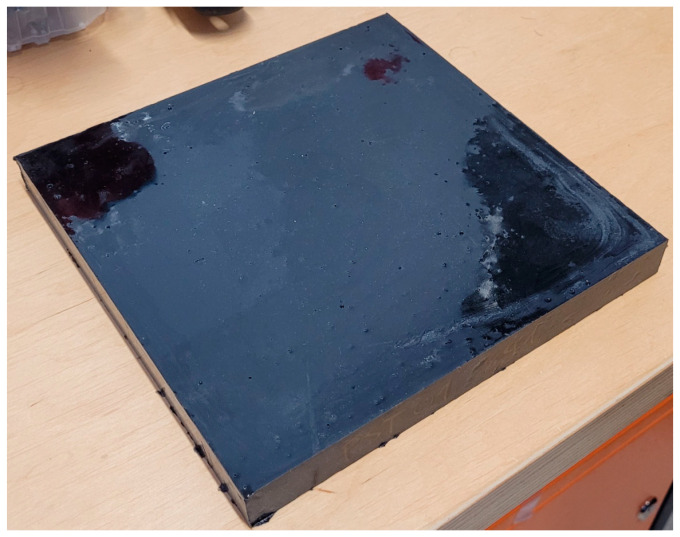
View of cuboidal sample (PST) showing surface of sample with irregularities.

A view of the sample with irregularities is shown in Figure 7. The heights of the cylindrical samples were chosen to mimic the irregularities of the cuboid samples, though on a smaller scale. This approach was motivated by the aim to examine the impact of even the smallest irregularities on measurement results.

### 2.3. FEM Model

Based on the data obtained from measurements of cylindrical samples, i.e., spring stiffness, the Young’s modulus values for the tested materials were estimated. With information on the material’s density and its Poisson’s ratio declared by the manufacturer, a complete set of data is available to prepare a FEM model using 3D elements and linear mechanics. This model aimed to estimate the dynamic stiffness of the tested cuboid samples under the assumption of ideal adhesion of the pressure plate (without irregularities).

A FEM model was prepared to estimate dynamic stiffness due to the stiffening effect in materials with flat geometry and a high Poisson’s ratio. The model was prepared using COMSOL Multiphysics software (v5.6). Using 3D geometry, the model replicated the test stand for dynamic stiffness previously described in this article [63]. The sample tested in the FEM model is a cuboid with dimensions of 200 mm × 200 mm × 20 mm. Material parameters for the model are shown in Table 5.

**Figure 8:**
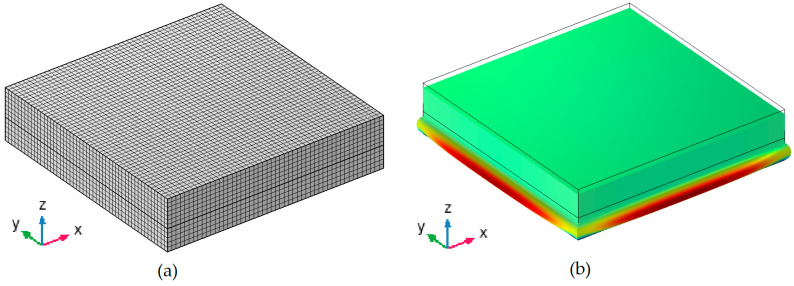
(**a**) Mesh of modelled dynamic stiffness bench test with cuboidal sample. (**b**) Mode shape of piston-like work of test bench.

The model, along with the finite element mesh and the evaluated mode shape for further analysis, is shown in Figure 8.

At the bottom of the sample, translation in all directions was constrained (prescribed displacement for each direction set to zero). The contact between the sample and the ballast plate was assumed to be ideal, without slip. All elements of the test setup were modeled as solid elements using hexahedral finite elements. The resonant frequencies of the modeled system were obtained from an eigenfrequency analysis. Based on the eigenfrequency corresponding to the piston-like mode shape (the one observed in the actual machine), the dynamic stiffness of the ideally adhered sample was determined.

## 3. Results

This section presents the measurement results of the resonant frequency (f_r_) and critical damping factor (CDF) for cuboidal and cylindrical samples. These values are obtained directly from measurements using the methods described in Section 2.

Table 6 shows the results for cuboidal samples. The resonant frequency is provided as average values, with the 95% credibility interval (95% CI) shown in parentheses. It was not possible to display the results for the critical damping factor due to limitations of the half-power bandwidth method. When the critical damping factor exceeds approximately 0.3, the results show limited sensitivity to parameters set in the data pre-processing algorithm, such as the smoothing method, degree of response curve, and background noise removal.

In Table 7, the average results for the resonant frequency and critical damping factor for sets of cylindrical samples across three different batches are presented. Compared to the results obtained from cuboidal samples tested for this paper, cylindrical samples exhibit significantly fewer issues related to pre-processing. The results of resonant frequency are stable and repeatable, and the critical damping factor is easily estimated.

## 4. Discussion

This section presents a discussion and analysis of the results concerning several aspects. The first aspect covers the theoretical normal pressure applied to the sample and the estimation of single cylinder stiffness. Next, the topics of Young’s modulus evaluation and the issue of sample irregularities in the context of dynamic stiffness are addressed. Practical consequences of the instability in dynamic stiffness results for the sound reduction index of building partitions are also highlighted. An attempt is made to predict errors in dynamic stiffness estimation using laboratory tests. Finally, the Rayleigh damping of cylindrical sample sets is analyzed.

This study stands apart from prior research by addressing the impact of sample irregularities and geometry on the accuracy of dynamic stiffness measurements. Unlike existing studies that assume idealized testing conditions [17,69,84,85,86], our work highlights the significant role of even small irregularities (0.19–1.13 mm) and demonstrates the advantages of cylindrical sample geometry in reducing measurement variability. Additionally, this study uniquely combines finite element modeling with experimental testing to validate these findings, filling a critical gap in the understanding of how real-world sample imperfections affect material characterization. These insights contribute to more accurate modeling and testing protocols for vibration and noise control materials.

### 4.1. Theoretical Normal Pressure Applied to Sample and Single Cylinder Stiffness Estimation

**Figure 9:**
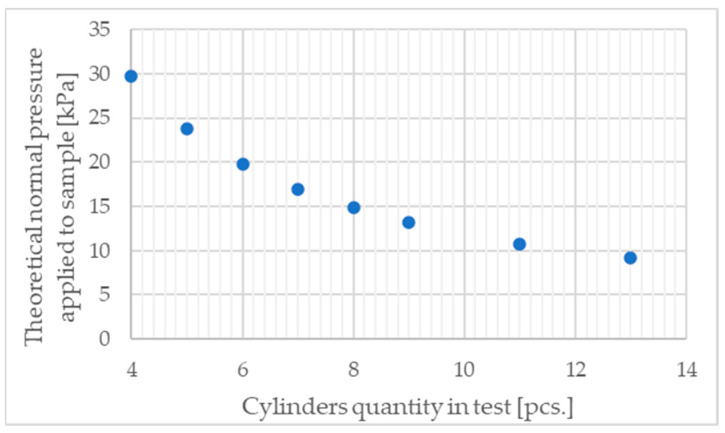
Theoretical normal pressure applied to cylindrical samples during test.

To calculate the theoretical normal pressure applied to the sample in the test stand, it was assumed that the samples have identical heights and that the bases of the tested cylinders are perfectly parallel to the extrusion direction. In brief, it was assumed that the cylinders are ideal. This assumption allows for drawing conclusions based on such a simplification. Adopting the actual geometry of the cylinders for calculations leads to a significantly more complex analysis due to the need to account for all geometric imperfections. The values of theoretical pressure applied to the samples by the test stand’s compression plate are presented in Figure 9.

Based on the measured resonant frequency of the cylindrical sample systems, the spring stiffness for a single cylindrical sample was estimated. The calculation of the total (equivalent) spring stiffness of the entire sample system was performed using Equation (5)

(5) $$f_{r}=\frac{1}{2\pi }\sqrt{\frac{k_{tot}}{m}}$$

where *f_r_* is resonant frequency, *k_tot_* is total (equivalent) spring stiffness, and *m* is spring supported mass in test (8 kg).

It was then assumed that each sample acts as a single spring connected in parallel with the others. This allows for determining the single spring stiffness (*k_i_*) (single cylinder stiffness) according to Equation (6):

(6) $$k_{tot}=\sum_{i=1}^{n}k_{i}$$

**Figure 10:**
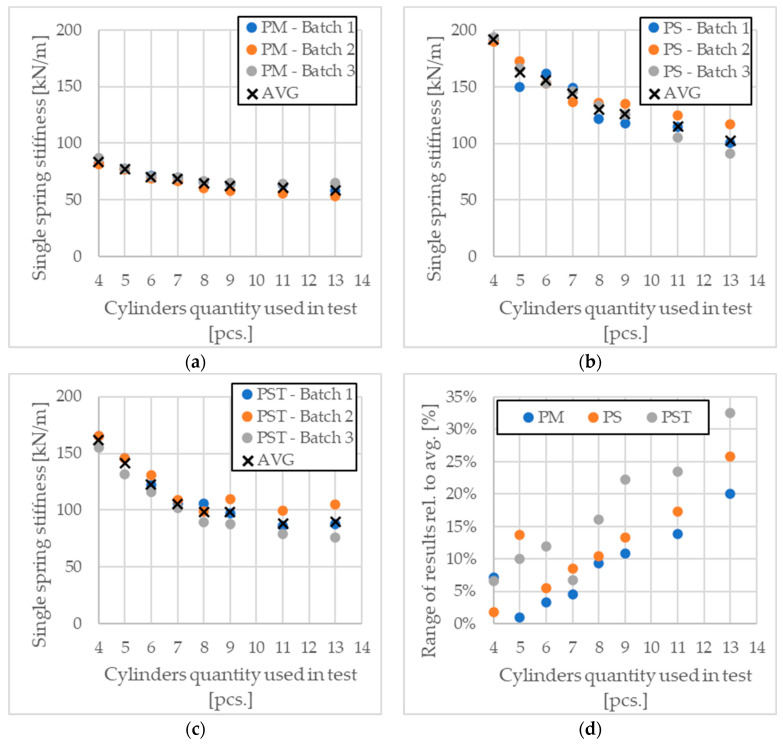
Results of single spring stiffness depending on the number of samples involved in the test (**a**) for PM, (**b**) for PS, and (**c**) for PST. (**d**) Range of results relative to average value.

By conducting the analysis based on the above equations and the results obtained for the cylindrical samples, the single spring stiffness can be determined. This operation was performed for all sample sets and batches. The results are presented in Figure 10.

Based on the analysis of the results shown above (Figure 10), several trends can be observed. In all materials, as the number of samples in the test increases (and consequently, as stress in each sample decreases), the single cylinder stiffness also decreases. Moreover, the range of results grows almost linearly for each material. The stiffness reduction with an increasing number of samples in the test is approximately 46% for PS, 45% for PST, and 30% for PM. The spread of results relative to the mean for a test with 13 samples reaches about 25% for PS, nearly 35% for PST, and 20% for PM.

While softer materials generally exhibit smaller variability in results, this trend is not strictly observed for PST compared to PS. PST, despite being softer based on the average Young’s modulus, shows greater variability in results (35%) compared to PS (25%). This suggests that additional factors, such as sample preparation or testing conditions, may also influence the spread in results.

### 4.2. Young’s Modulus Evaluation and Sample Irregularities Issue in the Context of Dynamic Stiffness

To estimate the Young’s modulus for the material, one can assume that a set of four cylindrical samples is the least sensitive to errors caused by sample irregularities. This assumption is based on the fact that the highest normal stress is present in these samples, leading to the greatest deformation in this direction. Consequently, sample irregularities play the smallest role in this case. The Young’s modulus estimation can be performed using the following procedure [84].

Based on the resonant frequency of the system, the static vertical deformation of the samples can be estimated.

(7) $$f_{r}=\frac{1}{2\pi }\sqrt{\frac{g}{e_{z}h}}$$

where *e_z_* is vertical strain, *g* is gravitational acceleration (9.81 m/s^2^), and *h* is sample height. In the next step, estimation of the apparent Young’s modulus (*E_a_*) from Hook’s law is possible.

(8) $$\sigma_{z}=E_{a}e_{z}$$

Finally, using the Equation (9), the Young’s modulus can be calculated, based on the *E_a_* values calculated from the measurements, Poisson ratio, and shape factor of cylindrical sample (*S* = r/h, r—is sample radius).

(9) $$\frac{E_{a}}{E}=\frac{1+3\nu \left(\frac{1-\nu }{1+\nu }\right)S^{2}}{1+3\nu \left(1-2\nu \right)S^{2}}$$

The results of forementioned procedure are used in the FEM model to obtain dynamic stiffness. The results of this analysis and comparison with measurements of the cuboidal samples dynamic stiffness are shown in Table 8.

The observed discrepancies between DS values obtained from FEM simulations and cuboidal sample measurements can be attributed to several factors. FEM simulations assume idealized boundary conditions, including perfect adhesion between the sample and testing plates, which are difficult to achieve in experimental setups. Experimental measurements with cuboidal samples are also influenced by surface irregularities (0.19–1.13 mm), which result in an apparent softening effect that reduces the DS values. Additionally, cuboidal samples exhibit more variability in stress distribution across the sample surface compared to cylindrical samples, further contributing to the inconsistency in measured DS values. These discrepancies emphasize the importance of accounting for realistic boundary conditions and minimizing surface irregularities to improve the correlation between experimental and numerical results.

**Figure 11:**
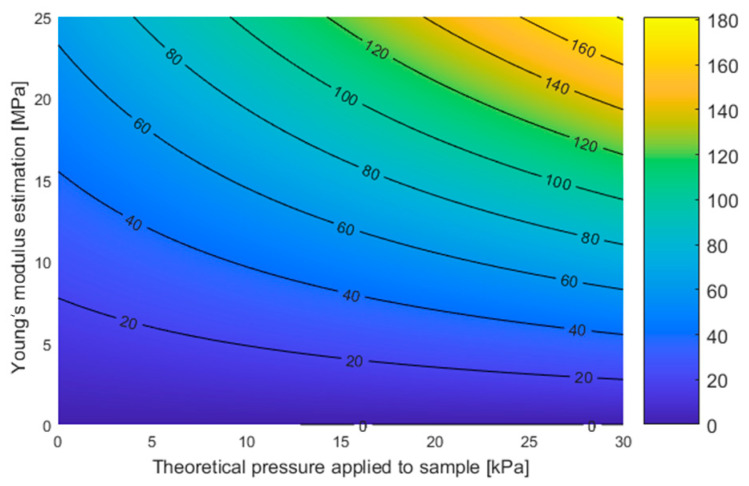
The estimated value of the spring stiffness of a single sample (*Z*-axis in [kN/m]) is presented as a function of the estimated Young’s modulus based on the set with four cylinders and the theoretical stress in the samples for all sets. (F(x,y) = 0.0180 − 0.0014 * x + 2.5760 * y + 0.1559 * x * y with R^2^ = 0.97).

The next part of the analysis will estimate the impact of material stiffness, expressed by the estimated Young’s modulus, on the final stiffness measurement results, assuming uniformly distributed stresses on the cylindrical samples. From previous information, it is evident that the stiffer the material, the more pronounced its apparent softening becomes. This effect can be attributed to sample irregularities (with a 95% credibility interval width of 0.092 mm for PM, 0.037 mm for PS, and 0.055 mm for PST). The result of the plane fitting is shown in Figure 11.

**Figure 12:**
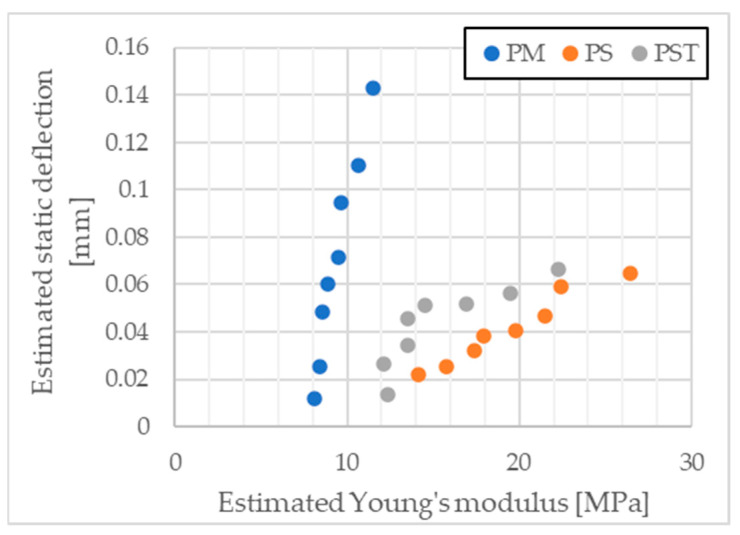
Estimated Young’s modulus from four cylindrical sample sets and static deflection estimated from resonant frequency (szerokość 95% credibility interval for PM = 0.092 mm, for PS = 0.037 mm, for PST = 0.055 mm).

With information on static deflection from the resonant frequency and the estimated Young’s modulus, the relationship between these two quantities can be examined. A summary of this relationship is shown in Figure 12.

From the above figures (Figure 11 and Figure 12), it can be inferred that as the Young’s modulus of the material increases and normal stresses decrease, the error in determining the stiffness of a single spring (cylindrical sample) also increases. This occurs when there are surface irregularities, simulated by cylindrical samples with small height differences, which are significant in the context of static deflection. In reality, not all cylinders experience the same normal stress.

### 4.3. Dynamic Stiffness Influence on Sound Reduction Index for Building Partitions

**Figure 13:**
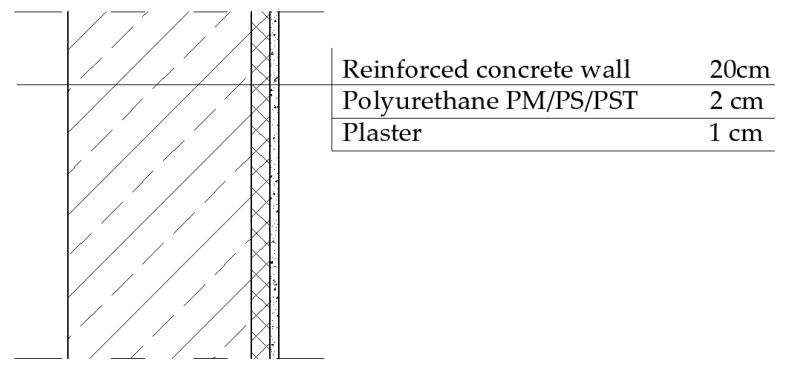
Reinforced concrete wall with additional layer of polyurethane and plaster.

Sample irregularities during testing can lead to an apparent softening of the material (lowering the dynamic stiffness result obtained from measurement). The estimated Young’s modulus or dynamic stiffness result may introduce errors in design. An example of such an error, due to improper estimation of dynamic stiffness, is the incorrect calculation of changes in airborne sound insulation of a building partition with an added layer. To illustrate the impact of this error, a wall made of reinforced concrete (density of 2400 kg/m^3^, thickness of 20 cm) with an additional polyurethane layer, as discussed in this article, and a plaster coating (density of 1700 kg/m^3^, thickness of 1 cm) was analyzed. The schematic is shown in Figure 13.

The change in airborne sound insulation was calculated according to the relevant standard EN 12354-1 [67] using the following relationship (Equation (10))

(10) $$f_{r}=160\sqrt{DS\left(\frac{1}{m_{1}^{\prime }}+\frac{1}{m_{2}\prime }\right)}$$

where *DS*—dynamic stiffness of the insulation layer [MN/m^3^], *m*_1_′—mass per unit area of the basic structural element [kg/m^2^], and *m*_2_′—mass per unit area of the additional layer [kg/m^2^]. Sound reduction index improvement ΔR_w_ is obtained from Table 9.

It should be noted that a negative value indicates a decrease in sound insulation, while a positive value indicates an increase in airborne sound insulation. The summary of results is presented in Table 10.

It would therefore be worthwhile to attempt to determine the threshold value of dynamic stiffness at which measurements can be considered reliable, or at least within an acceptable margin of error. To this end, an effort was made to establish a correlation between FEM-simulated dynamic stiffness for cuboid samples and the results obtained from laboratory tests on cuboid samples.

### 4.4. Prediction of Error on Dynamic Stiffness Estimation Using Laboratory Test

**Figure 14:**
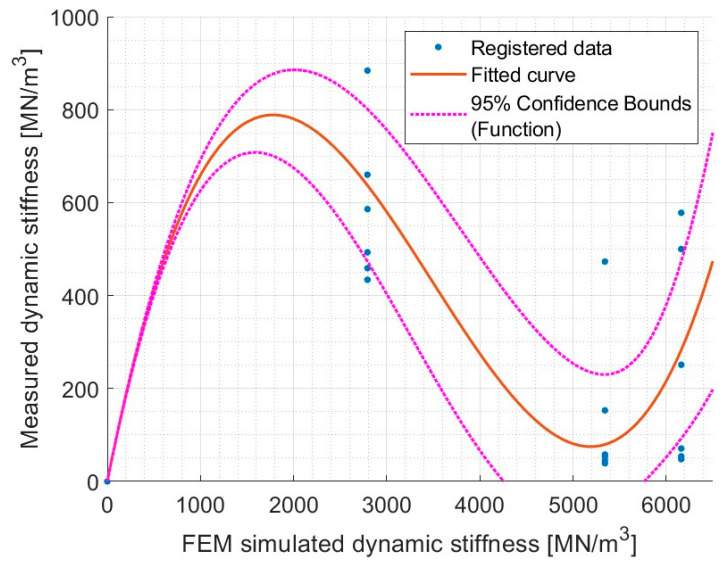
Correlation between FEM simulated dynamic stiffness and laboratory measured values of dynamic stiffness for cuboidal samples. val(x) = 3.607 × 10^−8^ * x^3^ + −0.0003771 * x^2^ + x, with R^2^ = 0.5387.

Based on the measured dynamic stiffness data for cuboid samples and known dynamic stiffness results from FEM analysis, an attempt can be made to predict the error for samples with a given Young’s modulus and irregularities within the statistical range of 0.19–1.13 mm (see Figure 14).

Due to the high variability in the results of measured dynamic stiffness in the lab test, the R^2^ value is relatively low but still above 0.5. Based on these data and correlations, an attempt can be made to estimate the expected percentage error associated with given surface irregularities on cuboidal samples. This summary is presented in Table 11.

### 4.5. Rayleigh Damping of Cylindrical Samples Sets

The analysis of damping data for the tested cylindrical sample systems reveals certain trends [88,89,90]. Unfortunately, a similar analysis for cuboidal samples is not possible, as only the resonant frequency could be determined using the available methods.

This analysis focuses on identifying the relationship between resonant frequency (*f_r_*) and the critical damping factor (*CDF*). This relationship enables us to determine whether the tested system can be described using Rayleigh damping as defined by Equation (11).

(11) $$CDF=\frac{1}{2}\left(\frac{\alpha }{2\pi f_{r}}+2\pi \beta f_{r}\right)$$

**Figure 15:**
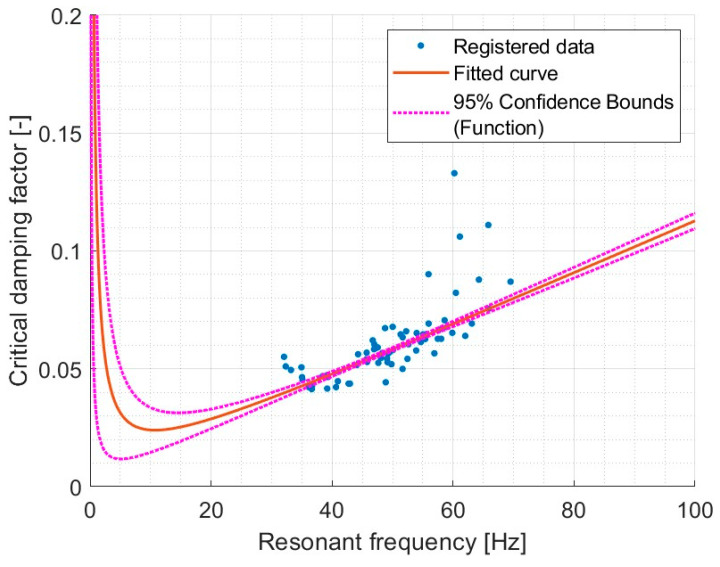
Model val(x) = 1/2 * (a * x + b/(x)) for all materials combined. For model coefficients see Table 12.

Initially, an attempt was made to find a general relationship between the critical damping factor and the resonant frequency. The purpose was to check whether the polyurethanes in the tested system follow the dependency described by Rayleigh damping. The results of the correlation analysis are presented in Figure 15.

An R^2^ value of 0.9754 indicates a very strong relationship between the critical damping factor and resonant frequency. This allows for determining the damping coefficients proportional to stiffness and mass. For a more detailed analysis, a summary of the Rayleigh damping model fit was prepared.

**Figure 16:**
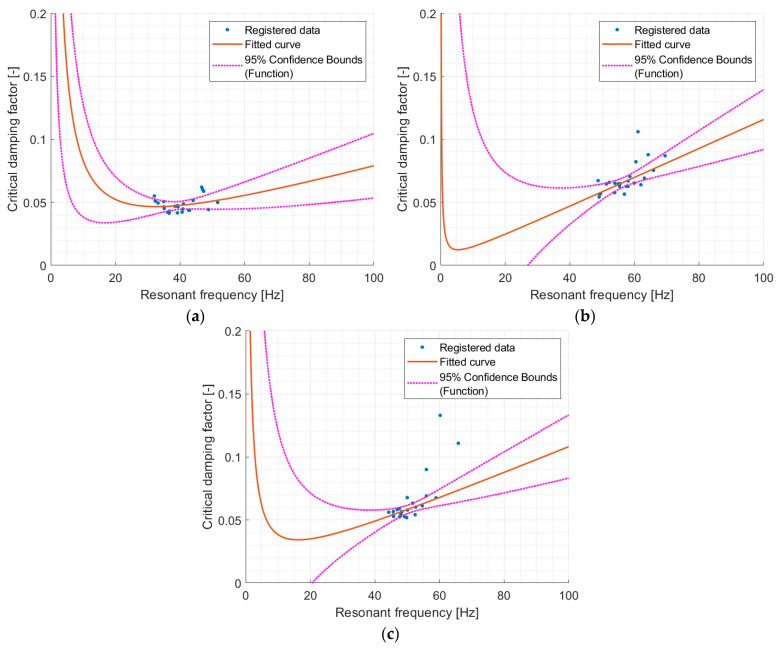
Model val(x) = 1/2 * (a * x + b/(x)) for Rayleigh damping for different materials: (**a**) PM, (**b**) PS, (**c**) PST. For model coefficients see Table 12.

As Figure 16 shows, for different materials, varying fits were obtained due to the differing ranges of resonant frequencies observed in testing and, most importantly, due to the distinct characteristics of each material. An interesting observation is the notably lower R^2^ value of only 0.06 for the PM material.

In general, it can be assumed that the sample system follows the relationship described by Rayleigh damping. However, for PM and PS, there is some uncertainty due to the relatively low R^2^ values (especially for PM), which makes it unclear whether this relationship can be considered reliable. Nonetheless, it is evident that for this type of polyurethane, there is a general dependency between the critical damping factor and resonant frequency.

## 5. Conclusions

In this article, laboratory and numerical studies were conducted to analyze the material properties of polyurethanes PM, PS, and PST. The research identified limitations of the dynamic stiffness testing method due to sample irregularities (0.19–1.13 mm) and proposed ways to mitigate this issue.

Irregularities in the range of 0.19–1.13 mm caused an apparent softening of the tested sample, resulting in measurements that are underestimated relative to the actual value. Furthermore, the results exhibit increased variability. The significance of irregularities grows with increasing material stiffness (Young’s modulus) or decreasing vertical deformation. However, the observed lack of a strong correlation between the stiffness of cylindrical materials and the standard deviation or spread in results indicates that this approach does not completely resolve the challenges of dynamic stiffness determination caused by sample irregularities. While cylindrical geometries mitigate some variability, particularly in cases of minimal irregularities, further research is required to refine the methodology and explore additional techniques for reducing error in dynamic stiffness measurements. These findings underline the need for careful consideration of sample geometry and preparation when assessing material properties.

It was observed that as the Young’s modulus of the material increases, the apparent error due to irregularities also rises. For an irregularity range of 0.19–1.13 mm and dynamic stiffness around 130 MN/m^3^, the error is about 5%, while for a dynamic stiffness of approximately 270 MN/m^3^, the error reaches 10%.

Even with relatively small, simulated irregularities, using cylindrical samples with slight variations in length (0.092 mm for PM, 0.037 mm for PS, and 0.055 mm for PST), the phenomenon of apparent softening in the tested sample system is still observed. However, for determining material properties, testing on cylindrical samples (especially with the minimum number of four samples) allows for a more reliable determination of dynamic stiffness than testing on cuboidal samples.

For the cylindrical sample systems studied, Rayleigh damping coefficients can be determined. This relationship is present across all types of material with cylindrical samples, as well as for individual materials. It is also observed that for rigid materials, as the resonant frequency increases, there is a corresponding rise in damping that deviates from the established trend.

## 6. Further Studies

An in-depth analysis of the increase in damping observed with rigid samples in the presence of irregularities raises important questions. Currently, it is uncertain whether the elevated damping results from errors in the half-power bandwidth method, when resonant frequencies are very close to one another, or if another mechanism might be responsible for this effect. This phenomenon could be highly beneficial for reducing the impact of vibrations on humans by increasing damping in structural systems.

The analyses in this article demonstrated that even slight irregularities in samples can significantly affect measurement results, which implies that conclusions drawn from such measurements may be prone to error. A more thorough investigation that definitively links the degree of irregularity with Young’s modulus, dynamic stiffness, and damping would enable a more informed approach to designing vibration isolation systems.

## Figures and Tables

**Table 1:** Machine parameters used for dynamic stiffness and damping evaluation [17].

Device Name/Manufacturer	Key Feature	Key Value of Parameters
Dynamic exciter—Brüel & Kjær (Virum, Denmark) Mini-shaker Type 4810	Provides sinusoidal force	Sine peak max 10 N Frequency range DC-18 kHz
Force sensor—Forsentek (Shenzhen, China) FSSM 50 N	Measures force applied to system	Max force 50 N Rated output 2.0 mV/V Hysteresis ± 0.1% R.O. (rated output)
IEPE accelerometer—MMF (Radebeul, Germany) KS78B.100	Measures acceleration of system response	Peak acceleration 60 g (~600 m/s^2^) Linear frequency range (5% deviation) 0.6 Hz–14 kHz
Dynamic stiffness test bench	Measures resonant frequency of sample set under load of 8 kg	Linear frequency range upper limit (5% deviation) 20–350 Hz—measured

**Table 2:** Materials used in tests.

Material Type (Manufacturer)	Nominal Density [kg/m^3^]	Poisson Ratio
Polyurethane PM (FlexAndRobust Systems, Cracow, Poland)	970	0.48
Polyurethane PS (FlexAndRobust Systems, Cracow, Poland)	1450	0.48
Polyurethane PST (FlexAndRobust Systems, Cracow, Poland)	1100	0.48

**Table 3:** Summary of sample data for cylindrical and cuboidal type.

Parameter	Material Type		
	PM	PS	PST
Cylindrical samples			
Mass [g]	59.0511 (59.0230; 59.0792)	88.2842 (88.1675; 88.4009)	68.0833 (67.9656; 68.2011)
Diameter [mm]	29.0467 (29.0334; 29.0600)	29.0517 (29.0433; 29.0600)	29.0653 (29.0521; 29.0784)
Height [mm]	91.8710 (91.8251; 91.9170)	91.9803 (91.9617; 91.9990)	92.0426 (92.0151; 92.0700)
Cuboid samples			
Mass [g]	794.00 (779.08; 808.92)	1186.67 (1148.63; 1224.71)	904.33 (877.85; 930.82)
Height [mm]	19.8883 (19.5975; 20.1791)	20.4917 (20.1622; 20.8211)	19.7700 (19.1585; 20.3815)

**Table 4:** Irregularities of cuboid samples.

Parameter	Material Type		
	PM	PS	PST
Irregularities with 95% credibility interval in brackets [mm]	0.89 (0.22; 1.13)	0.41 (0.21; 0.73)	0.60 (0.19; 0.75)

**Table 5:** Material parameters used in FEM model.

Element	Density [kg/m^3^]	Poisson Ratio [-]	Young’s Modulus [GPa]
Steel plate	7850	0.30	210
Test sample (PS, PM, PST)	As in Table 2 PS, 1450 PM, 970 PST, 1100	As in Table 2 (0.48)	From analysis (see Section 4.2)

**Table 6:** Results for cuboidal samples.

Material Type	f_r_ [Hz] (95% CI)	CDF [-]
PM	270.28 (230.92; 309.63)	beyond method capabilities (>0.3)
PS	159.26 (67.76; 250.76)	beyond method capabilities (>0.3)
PST	116.49 (45.60; 187.38)	beyond method capabilities (>0.3)

**Table 7:** Results for cylindrical samples.

Material Type	Cylinders Quantity [pcs.]	f_r_ [Hz]			CDF [-]		
		Test Batch 1	Test Batch 2	Test Batch 3	Test Batch 1	Test Batch 2	Test Batch 3
PM	4	32.3	32.1	33.2	0.0510	0.0551	0.0495
	5	35.0	34.9	35.1	0.0465	0.0506	0.0452
	6	36.7	36.1	36.6	0.0431	0.0422	0.0414
	7	39.4	38.5	39.2	0.0465	0.0469	0.0474
	8	41.0	39.2	41.1	0.0447	0.0416	0.0487
	9	42.7	40.7	42.9	0.0437	0.0422	0.0437
	11	47.0	44.1	47.3	0.0604	0.0516	0.0588
	13	48.9	46.7	51.6	0.0443	0.0621	0.0500
PS	4	49.3	49.1	49.5	0.0565	0.0543	0.0571
	5	48.8	52.3	51.3	0.0673	0.0659	0.0646
	6	55.4	54.0	53.9	0.0626	0.0652	0.0578
	7	57.5	55.0	56.9	0.0627	0.0646	0.0566
	8	55.6	58.6	58.1	0.0648	0.0706	0.0669
	9	58.0	62.0	59.9	0.0627	0.0640	0.0653
	11	63.1	65.9	60.5	0.0692	0.0754	0.0822
	13	64.3	69.5	61.1	0.0878	0.0870	0.1061
PST	4	45.7	45.8	44.3	0.0568	0.0529	0.0562
	5	48.1	48.1	45.7	0.0552	0.0546	0.0541
	6	48.1	49.8	46.9	0.0559	0.0519	0.0583
	7	48.3	49.2	47.5	0.0568	0.0528	0.0591
	8	51.7	50.0	47.6	0.0633	0.0678	0.0525
	9	52.6	56.0	50.1	0.0604	0.0692	0.0580
	11	54.7	58.9	52.5	0.0614	0.0677	0.0542
	13	60.2	65.8	56.0	0.1330	0.1110	0.0901

**Table 8:** Results of FEM analysis with recalculation of Young’s modulus from cylindrical samples.

Material Type	Cylinders Quantity [pcs.]	Average Young’s Modulus [MPa]	FEM Estimated f_r_ [Hz]	DS from FEM Simulations [MN/m^3^]	DS from Cuboid Sample Measurements (95% CI) [MN/m^3^]
PM	4	11.51	594.73	2793	421–757
	5	10.67	572.65	2589	
	6	9.66	544.67	2342	
	7	9.46	539.03	2294	
	8	8.88	522.47	2155	
	9	8.57	513.23	2080	
	11	8.42	508.59	2042	
	13	8.07	498.04	1958	
PS	4	26.42	883.45	6162	36–497
	5	22.45	814.44	5237	
	6	21.47	796.60	5010	
	7	19.82	765.45	4626	
	8	17.93	728.06	4185	
	9	17.39	717.10	4060	
	11	15.79	683.31	3687	
	13	14.16	647.14	3307	
PST	4	22.28	822.60	5343	16–277
	5	19.44	768.57	4664	
	6	16.91	716.85	4057	
	7	14.52	664.43	3486	
	8	13.48	640.22	3236	
	9	13.55	641.77	3252	
	11	12.14	607.68	2916	
	13	12.36	613.17	2969	

**Table 9:** Weighted sound reduction index improvement by an additional layer, depending on the resonance frequency [67].

Resonance Frequency of the Additional Layer [Hz]	ΔR_w_ [dB]
<80	35—R_w_/2
100	32—R_w_/2
125	30—R_w_/2
160	28—R_w_/2
200	−1
250	−3
315	−5
400	−7
500	−9
630 to 1600	−10
>1600	−5

**Table 10:** Comparison of dynamic stiffness estimation from lab test of cuboidal samples and FEM analysis.

Data Source	Material Type	DS [MN/m^3^]	f_r_ [Hz]	ΔR_w_ [dB]
Cuboidal sample test	PM (95% CI)	421	810	−10
		757	1086	−10
	PS (95% CI)	36	237	−3
		497	880	−10
	PST (95% CI)	16	158	0
		277	657	−10
FEM analysis with data from cylindrical sample test (4 pcs. batches)	FEM results for PM, PS, PST (min, max)	1958	1747	−5
		6162	3100	−5

**Table 11:** Comparison of dynamic stiffness estimation from lab test of cuboidal samples and FEM analysis.

Relative Error	1%	5%	10%	50%
Measured Value	26.73	127.32	244.83	779.05
FEM simulated value based on cylindrical samples	27	134	272	1558

**Table 12:** Models’ coefficients for Rayleigh damping of all materials.

Material Type	Model Coefficients		Rayleigh Damping Coefficients		R^2^
	a	b	α	β	
All materials	0.002231	0.251	0.01402	0.03995	0.9754
PM	0.001429	1.522	0.00898	0.24223	0.0581
PS	0.002309	0.06631	0.01451	0.01055	0.6720
PST	0.002109	0.5587	0.01325	0.08892	0.9075

## Data Availability

The original contributions presented in the study are included in the article, further inquiries can be directed to the corresponding author.
